# Conflicts – Oil Exploration and Water

**DOI:** 10.1002/gch2.201600015

**Published:** 2017-07-13

**Authors:** Nenibarini Zabbey, Gustaf Olsson

**Affiliations:** ^1^ Department of Fisheries, Faculty of Agriculture University of Port Harcourt PMB 5323, East‐West Road Choba Rivers State Nigeria; ^2^ Department of Biomedical Engineering Lund University Box 118 SE‐22100 Lund Sweden

**Keywords:** deepwater horizon, leakage detection, Niger Delta, oil accidents, oil exploration, oil tanker

## Abstract

Water resources and water quality are closely related to oil exploration, refining and distribution. Since oil products provide over 90% of transport energy in almost all countries it is apparent that any oil operation is an inherent risk for water resources. Since water supplies will be increasingly stressed as a consequence of climate change and population increase the environmental risks associated with oil exploration may intensify. Thus, there are more reasons than CO_2_ emissions and climate change to cut down on oil production and consumption. In this paper water related risks are discussed from two aspects: (1) water use and water pollution as a result of normal exploration, refining and distribution, (2) water and marine life contamination caused by accidents. It will be exemplified by some major oil accidents, too often caused by human errors or negligence. Ecological effects of oil contamination for seawaters and freshwaters are discussed. Some aspects of social and economic consequences are examined. Some possibilities for mitigating oil leakage risks are highlighted.

## 1 Introduction

Water related risks in oil exploration, refining and distribution are discussed in this paper. Since oil products provide over 90% of transport energy in almost all countries, oil supply interrupts may have serious effects, not only on mobility, but also on food production and distribution, heating, medical care, national security, manufacturing, and other vital functions of modern societies. When oil is explored in water scarce areas the water resources become stressed. Off‐shore oil exploration creates risks for the marine life while oil distribution and transportation will generate increasing risk for the ecology in case of leakages or accidents. We will illustrate the risks by some examples of accidents and mishaps that have affected water resources and water quality.

The petroleum industry generally classifies crude oil by the geographic location it is produced in, its density, and its sulfur content. A light crude oil has a low density and a heavy crude oil has a high density. An oil well produces predominantly crude oil, with some natural gas dissolved in it. Because the pressure is lower at the surface than underground, some of the gas will come out of solution and will be either recovered or burned.

Crude oil is also found in semi‐solid form mixed with sand and water, as in the Athabasca oil sands^1^ in Canada, where it is usually referred to as crude bitumen. Bitumen is a sticky, black, tar‐like form of crude oil which is so thick and heavy that it must be heated or diluted before it will flow.

Shale gas is natural gas that is trapped within shale formations. Shales are fine‐grained sedimentary rocks that can be rich sources of petroleum and natural gas. Drilling technology development in combination with hydraulic fracturing (“fracking”) has made it possible to extract the shale gas and oil at competitive prices.

It is well established that fossil‐fuel combustion accounts for most of the global CO_2_ emissions. However, climate change is not the only reason to cut oil production. Two factors that relate oil to water are considered here: (1) water pollution as a result of normal exploration, refining and distribution, (2) water and marine life contamination caused by accidents. Since water supplies will be increasingly stressed in many regions as a result of climate change and population increase the environmental consequences of accidents and failures in oil production infrastructures will have a rising impact on society in many regions. Furthermore, as oil exploration may take place under very difficult operating conditions, for example deep below the sea floor, the risks for accidents with serious consequences seem to escalate.

The impact of fossil fuel on climate change is well recognized. The key findings are:^2^

- The potential carbon emissions from the oil, gas, and coal in the world's currently operating fields and mines would take us beyond 2 °C of warming.
- The reserves in currently operating oil and gas fields alone, even with no coal, would take the world beyond 1.5 °C.
- With the necessary decline in production over the coming decades to meet climate goals, clean energy can be scaled up at a corresponding pace, expanding the total number of energy jobs.

IPCC expresses possible future global warming in a different way^3^ and defines several Representative Concentration Pathways (RCP), expressed as scenarios of human activities. The four RCPs include one mitigation scenario leading to a very low forcing level (RCP2.6), two stabilization scenarios (RCP4.5 and RCP6), and one scenario with very high greenhouse gas emissions (RCP8.5). The findings are summarized as: **“**Global surface temperature change for the end of the 21^st^ century is likely (66–100% probability) to exceed 1.5 °C relative to 1850 to 1900 for all RCP scenarios except RCP2.6. It is likely to exceed 2 °C for RCP6.0 and RCP8.5, and more likely than not (>50–100%) to exceed 2 °C for RCP4.5. Warming will continue beyond 2100 under all RCP scenarios except RCP2.6.”

In the recent World Energy Outlook^4^ it is found that there will still be difficulty to find alternatives to oil in road freight, aviation and chemicals before 2040. However, the number of electric cars will increase globally, from 1.3 million in 2015 to over 30 million by 2025 and more than 150 million by 2040, according to the New Policies Scenario. This will displace 1.3 mb d^–1^ (million barrels per d. 1 mb d^–1^ = 0.159 million m^3^.) of oil. Still, additional policy support could result in as much as 710 million electric cars in 2040. This would displace more than 6 mb d^–1^ of oil demand. In 2015 the world oil demand was 93 mb d^–1^. The New Policies Scenario predicts the demand to increase to more than 103 mb d^–1^, while in the more progressive 450 scenario the global oil demand peaks by 2020, at just over 93 mb d^–1^. The demand by 2040 then is predicted to 73 mb d^–1^.

It is obvious that the continued global oil demand will require huge oil explorations and there are reasons to believe that the environmental risks will grow when new sources of oil are explored. The carbon footprint of fossil fuels is not considered here. However, water is most often the first casualty of a faulty operation or accident.

## 2 Water Related Risks in Oil Exploration

Accidents, leakages and spills are realities in oil drilling, shale gas exploration, pipeline transportation as well as in oil tanker transportation. Some of the impacts on water resources and water quality are described here. A more detailed account is provided in ref. 5.

### 2.1 Drilling for Crude Oil

Water is needed for the extraction of oil from underground sources as well as for the refining of the crude oil. Most new commercial oil and gas wells are initially free flowing, so that the underground pressures drive the liquid and gas up the well bore to the surface. To drill wells requires water for preparing drilling fluid: cleaning and cooling of the drill bit, evacuation of drilled rocks and sediments, and providing pressure to avoid collapse of the well. Drilling fluid contains potential contaminants.

Oil reservoirs frequently contain large volumes of water. In order to maintain the reservoir pressure it is common to inject gas, water, or steam into the reservoir. In some cases, the oil may be too heavy to flow. A second hole is then drilled into the reservoir and steam is injected under pressure. The heat from the steam thins the oil in the reservoir, and the pressure helps push it up the well. Today, most oil producers re‐inject produced water or reuse it for onshore wells (98%). However, 91% of produced water from offshore wells is discharged into the ocean.^6^

A typical drilling accident is caused by blowouts of liquid and gaseous hydrocarbons from the well when the operation has encountered zones with abnormally high pressure. A “blowout” occurs when a mixture of pressurized natural gas, oil, mud, and water escapes from a well, shoots up the drill pipe to the surface, expands and ignites. There are mainly two categories of drilling accidents:

- Catastrophic situations involving intense and prolonged hydrocarbon gushing. The pressure in the drilling zone is very high and the routine methods of well muffling (devices to reduce vibrations) do not help. This kind of extreme event is quite rare.
- Regular episodes of hydrocarbon spills and blowouts during the drilling operation. This mishap can be controlled by the help of blowout preventers (a massive stack of shut‐off valves and auxiliary equipment that sits on top of the well) and by changing the density of the drilling fluid. Usually these kinds of accidents do not get a lot of media attention. Still, there is a considerable ecological risk, primarily due to the regularity of the events. The logical consequence is a chronic impact on the marine environment.

The environmental consequences of accidental episodes are especially severe, sometimes dramatic, when they happen near the shore, in shallow waters, or in sheltered areas with slow water circulation.

### 2.2 Shale Gas Exploration

Oil extraction has traditionally been performed from conventional reservoirs. However, producers have long known shale as “source rock”—rock from which oil and natural gas slowly migrated into traditional reservoirs over millions of years. Shale gas is natural gas locked in layers of impermeable hard rock, shale formations. Oil shale deposits can be considered as an immature oil field.^7^

The development of two technologies has been decisive for the commercial success of shale gas. One groundbreaking technology in the early 1990s achievement was the combination of vertical with horizontal drilling, where the drill at depth (typically ≈3000 *m*) can be turned 90° to access horizontal shale layers where large amounts of natural gas and oil that are usually trapped can be released by shattering the shale. The other key technology to make shale gas economically feasible was the development of hydraulic fracturing. The dense rock simply had to be broken up in order to reach the trapped oil in the pores of the rock. The first experiments with hydraulic fracturing took place in 1947. Fracking will widen the existing cracks by pumping water mixed with proppants (mostly sand) and chemicals under high pressure. Hydraulic fracturing in combination with horizontal drilling made the U.S. shale gas ‘revolution’ possible.

Two principal water issues associated with fracking are:

- The use of a large amount of freshwater that becomes contaminated and which can never again be used by humans, animals or plants for any purpose, and
- the necessity of protecting underground water tables and surface water resources from contamination by fracking fluids and/or migrating gas deposits.

Some 0.5–2% (by volume) of the fracking fluid is composed of a blend of chemicals, often proprietary, that enhance the fluid's properties.^8^ Biocides and certain petroleum products that are present in fracturing fluid are particularly hazardous chemicals that may cause health risks that range from rashes to cancer.^9^ The chemical composition is highly variable and consequently the toxicity of the produced water will vary a lot.

A lot of attention has been directed toward the possibility of subsurface migration of fracturing fluids or hydrocarbons into groundwater aquifers.^10^ Low‐permeability natural gas resources are in geologic formations located at depths of 450–4500 m below the surface, with natural gas wells averaging 2000 m.^8^ At these depths, the formations may underlie drinking water aquifers, which are commonly 30–100 m below the surface. However, there are various risks related to the handling of the fracking fluid related to:

- leakages from the drilling;^11^
- handling of returned water–spills and accidents.^12^

As a consequence the influence from fracturing to drinking water sources is considered a real risk.

### 2.3 Oil Sand

Oil sand consists of layers of sticky, tarlike bitumen mixed with sand, clay and water. In many deposits some 30 m of soil must be stripped off to reach the oil sand. Oil sand surface mining operates on large scales. The sand is delivered to an extraction plant where the bitumen is separated from sand in a hot‐water wash, sometimes with caustic soda, to facilitate the separation of bitumen from solids. Most of the potentially recoverable bitumen is in deposits deeper than 60 *m*. When the bitumen is located too deep to be strip‐mined, the industry melts it in situ with copious amounts of steam, thus decreasing the bitumen viscosity, so that it can be pumped to the surface.

Large volumes of water are needed for extracting bitumen from the oil sands. The Canadian National Energy Board (NEB) has calculated that to produce 1 *m* ^3^ of synthetic crude oil 2–4.5 m^3^ of water is needed.^13^ Very little data concerning the fate of the wastewater contaminants have been released but some studies are reported.^14^

### 2.4 Pipelines

Oil pipelines made of steel rust from the water in the oil. When super‐heated water is used to blast the oil the water mixes with the dirt contained in the crude oil and forms sludge at the bottom of the pipe. This breeds microorganisms that can produce sulfur that will accelerate the corrosion process. Transporting tar sand faces additional challenges, since tar sand oil is much thicker than traditional oil. It contains 15–20 times higher acid concentrations and 5–10 times more sulfur than conventional crude oil. To transport it in pipelines requires that it is mixed with other components, some of which are hazardous.^15^

A so called pinhole leak of 2% is usually not detected by any instruments. If the leak appears in an uninhabited area it may not be noticed for months. Apparently such a leak with water‐soluble toxins can easily reach a river in rain or spring runoff. If the leak happens under water in a wetland it may not be noticed until the pipe bursts.

### 2.5 Transport on Sea

A database on ship source‐pollution is maintained by the International Tanker Owners Federation Ltd (www.itopf.com). Oil spills of over 700 tons, from oil tankers, ore carriers and tank barges are listed since 1967. The consequences of the spills are recorded as well as various aspects of the spills. In the period 1970–2000 5.5 million tons of oil has been spilled in the seas by oil tankers, an average of 0.18 million tons per year with a maximum of 0.64 million in 1979. There is a positive trend with less tanker spills, so from 1990 to 1999 1.14 million tons were spilled, where 73% were caused by 10 accidents.

According to insurance companies' statistics^16^ some 80% of oil tanker accidents causing oil spills at sea are a result of human errors. A high proportion of the spills are caused by groundings and collisions.

## 3 Major Oil Accidents

The water related risks of oil exploration are illustrated by some examples. The Mexican Gulf disaster in 2010 was caused by one major explosion on the oil drilling platform in the sea. The Niger Delta destruction has been caused by long term leakages from pipelines into fresh and brackish water wetlands. The examples of tanker disasters are used to illustrate the ecological risks related to sea transport of oil.

In this section we describe the magnitude of the spills and in the following section we briefly describe the ecological impact of the oil leakages. In the last section we discuss some social and economic consequences of the spills.

### 3.1 Mexican Gulf

The explosion of BP's Deepwater Horizon rig in April 2010 followed by the spill in the Mexican Gulf is officially the largest oil disaster ever. The accident killed 11 people. An oil well about 1500 m below the sea surface blew out causing an explosion on the platform. Somehow the blowout preventer failed. Two switches–one manual and one automatic backup–failed to start it. Oil leaked out from the well for 85 d until the well was finally capped on July 15, 2010. Altogether 780 000 m^3^ of oil leaked out into the sea, which corresponds to a daily average flow rate of more than 9000 m^3^. About 920 km of the Gulf shoreline was contaminated. Moreover almost 7000 m^3^ of dispersant was used causing harm on the fragile ecosystem. The disaster was widely reported in media, see e.g. ref. 17. Some of the water related consequences are discussed in next section.

### 3.2 Niger Delta, Nigeria

Unlike the Deepwater Horizon accident the oil spills in Nigeria are not caused by one major accident but are the result of thousands of spills and leakages during several decades. These spills have attracted much less attention in international media than the Mexican Gulf accident despite the fact that the accumulated oil spills in the Niger Delta are the largest in the world. International organizations and independent experts have estimated the extent of the damages.

Nigeria has Africa's largest reserves of oil and gas within its borders and most of these resources exist in the Niger Delta and on the continental shelf of the country.^18^ The oil deposits have been extracted since the 1950s. The petroleum industry in the Niger Delta accounts for over 90% of the country's total foreign exchange revenue.^19^

The geographic Niger Delta, approximately 26 000 km^2^ in size, is the third largest wetland and has the fourth largest mangrove area in the world. The Delta is home of extraordinary biodiversity (some of which are endemic) and is also endowed with several mineral deposits such as marble, barites, limestone, sand and gravel.^20^

Oil spills have occurred repeatedly for decades in the Niger Delta and large parts of the land are chronically affected by the spills. Tidal fluxes accelerate and exacerbate the effect of a leak by spreading oil further afield. Since the Niger Delta is one of the wettest areas on earth, floods in connection with high rainfall will rapidly distribute and remobilize spilt oil over large areas.^18^

Records between 1976 and 2001 alone indicate that 6817 oil spills occurred in the Niger Delta resulting in the loss of, approximately 500 000 m^3^ of oil,^21^ or almost 20 000 m^3^ per year. Between 1.4 and 2.1 million m^3^ of oil had been leaking out into the Niger Delta ecosystem over the past fifty years.^22^, ^23^, ^24^, ^25^

Oil spills are often under‐reported. The Nigerian Government and the oil companies operating in the Delta maintain their own data on leakages and the data are sometimes unreliable and conflicting as both the Government and operators seek to limit their legal liability for commensurate claims and compensations from oil spill damages.^22^, ^26^ In worst cases, oil spillages in the Delta are never reported or merely branded minor without minimum post‐spill containment, recovery and remediation responses.^27^

Volume estimates of oil spills are usually low, since 50% of Nigerian oil is assumed to evaporate within 48 hours, and leakages are not usually detected in that period. Most spills in the Delta are left unattended. It has been documented that regulatory oversight of the petroleum industry in Nigeria and some industry practices, especially oil spills management, fall below acceptable international best practices.^20^, ^23^, ^26^, ^28^, ^29^, ^30^ As documented in a Nigerian Government report in 2006: “Oil companies operating in the Delta … can easily improve their environmental performance in the region. Old leaking pipelines and installations must be replaced immediately and dumping of waste must stop.”

Causes of oil contamination in the Niger Delta include discharges from nearshore operations; urban and industrial effluents discharge; ballast water from oil tankers; accidental spills during loading; equipment failure at loading sites; leaking pipelines, wellheads, and flow stations due to poor maintenance and corrosion; from illegal tapping of the wells (bunkering); and from artisanal refining under very primitive conditions.^18^, ^23^, ^27^

The Trans‐Niger Pipelines (TNPs), transporting crude oil from the hinterlands through Ogoni to Bonny crude oil terminal presents potential threats of oil spillages. The pipeline failure rate per 1000 km and year in 2004 was 6.40 in Nigeria, 15 times higher than the Western European value of 0.43.^26^ This is called “a double standard” in the report.^26^ The TNPs suffered an incidence of operational oil spills at a rate of more than 130 times greater than the European average in the period 2006–2010.^31^, ^32^

### 3.3 Oil Tanker Disasters

Tanker accidents have been reported extensively in both the scientific literature and the media. Although the rate of tanker accidents has been declining over the past two decades the disasters are naturally not unavoidable. Most of the attention to the damage is not paid towards the sea water quality but on how the levels of oil pollution have reached lethal limits for marine fauna, mainly for birds and mammals.

One of the largest disasters is the Amoco Cadiz accident in March 1978. The very large crude carrier was dragged by a storm off the coast of Brittany, France. The ship split in two parts and quickly sank before any oil could be pumped out of the wreckage and 1.6 million barrels (255 000 m^3^ or 220 000 tons) of oil were contaminating the sea waters. The disaster created an oil slick 30 km wide and 130 km long and polluting 320 km of coastline in the process. Although the clean‐up operation did manage to collect around 100 000 tons of oil and water, less than 20 000 tons of oil could be recovered from this liquid after treatment in refining plants.^33^

In March 1987 Torrey Canyon struck a reef near Land's End, Cornwall, southwestern UK, owing to a navigational error. Unsuccessful attempts were made to float the ship off the reef. The ship broke apart after being stranded on the reef for several days and was bombed by aircraft in attempts to burn off some of the oil in the tanker. Some 120 000 m^3^ (≈95 000 tons) of oil were spilled into the sea. Rescuers used napalm and petrol to try and burn off the oil on the sea's surface. The ship's entire cargo either ended up in the sea or was burnt off over the next twelve days. Around 80 km of the French and some 190 km of the Cornish coast were polluted, altogether 700 km^2^ of slick. Dispersants were used heavily to break up the slick and this caused great damage. These were first‐generation variants of products originally formulated to clean surfaces in ships' engine‐rooms. There was little concern over the toxicity of their components, and many observers believed that they were officially referred to as ‘detergents’. They were in fact ‘solvent‐emulsifiers’. Attempts to use foam booms to contain the oil were also of limited success due to their fragility in high seas. Vessels sprayed over 10 000 tons of the dispersants onto the floating oil and they were also deployed against oil stranded on beaches.^34^

The Exxon Valdez supertanker hit a reef off the Alaskan coast in 1989. Eleven of its cargo tanks ruptured, dumping 43 000 m^3^ of crude oil into Prince William Sound. The spill could have been much worse —the tanker was carrying 200 000 m^3^. Despite attempts to use dispersing agents and oil skimming ships, oil washed onto some 2000 km of Alaskan coastline.^35^ After decades, oil remains a few inches below the surface on many of Alaska's beaches.

In January 1993 the supertanker Braer encountered hurricane‐force winds off the Shetland Islands, Scotland and ran aground. Some 85 000 tons of crude oil was spilled. The clean‐up operation was severely hampered by a month‐long January North Sea storm that made access to the ship and the site difficult. However, the bad weather also helped to disperse the massive oil slick caused by the incident.^36^

## 4 Environmental Impact – Water Quality and Aquaculture

Oil leakages have long lasting effects on water quality and ecology, both in seawaters and in freshwater areas, as exemplified below from the warm waters in the Mexican Gulf, from cold waters in Alaska and from the Niger Delta.

### 4.1 Fate of the Oil in Sea Waters

A crucial question after an oil spill is where all the spilled oil will go. The fate of the oil and the movements of the various fractions of the oil have to be understood. The physical and chemical composition of the oil can change quickly and depend on weather and temperature.^37^ Hydrocarbon evaporation of surface spills will start immediately and most of it may take place in the first day. The size of the evaporation loss depends on the initial oil composition.^38^ Lighter components will be dissolved into the water from the underside of the slick.

For droplets from deep water spills there is a long travel time before they can reach the surface. Therefore dissolution is the dominating initial process as found in studies after the Ixtoc I platform accident in the Mexican Gulf in 1979, when 500 000 m^3^ of crude oil was spilled.^39^ After the Deepwater Horizon spill it was found that large volumes of oil appeared on the sea floor, which required further explanations for the mechanisms. One explanation has been dragdown by settling particles. Another explanation is that following the evaporation of the light constituents from the oil mixture the density of the residue will increase, thus causing the oil to sink.^40^

The question appears how long the impact of a spill can last. Ten years after the Ixtoc accident it was found that in a protected reef lagoon the Ixtoc tar mat was still partially buried in the sediments. On the ocean side of the reef, where winds and waves and currents are stronger, no oil remained. Where there is wave energy and oxygen, sunlight and microorganisms will degrade the oil. The bacteria feed on oil and methane and will deplete the water of oxygen. Since water in the deep sea mix very slowly oxygen depleted zones could persist for decades. When oil falls to the bottom and gets entrained in low‐oxygen sediments, as in a lagoon or in a marsh, it can remain for decades. Naturally this will have devastating consequences for the ecosystem.^41^

Another impact of the Mexican Gulf spill in 2010 is the heavy use of subsea dispersants like Corexit. Despite assurance from BP that Corexit was not toxic it was found that it was more toxic to marine life than the oil itself.^42^ On May 19, 2010 EPA instructed BP that the company had to immediately identify and use less‐toxic form of chemical dispersants.

The Exxon Valdez accident in 1989 occurred in cold water. Early experimental oil release field studies, in Arctic freshwater and marine ecosystems, and follow‐up studies after Arctic and subarctic oil spillages indicate long persistence times for the oil contaminants and slow microbial biodegradation.^43^ Ten years after Exxon Valdez it was reported that less than 15% of the spill was recovered.^44^ Most of the oil had evaporated or biodegraded. Sandy beaches are washed by waves. Sheltered from the surf, the oil can remain beneath and between rocks. Low temperatures alone cannot explain the limited rates of hydrocarbon biodegradation. Rather the limitation to microbial degradation of oil in Arctic ecosystems appears to be due to the combination of several factors, including the availability of nitrogen, phosphorus, and oxygen.^43^ In the Exxon Valdez case a nitrogen‐phosphorous fertilizer mix was sprayed on an oil‐laden shore in hopes of stimulating the microbial activity. Still the microbial hydrocarbon degradation rate is low in these waters and the time frame after a major spillage will be decades rather than years.^43^ There is a vast literature on the long term ecosystem response to the Exxon Valdez accident.^45^

### 4.2 Impact on Sea Marine Life

Oil has a huge impact on marine life. It is outside the scope and space for this paper to describe details. There are numerous accounts on how oil harms animals and plants in the marine environment. An overview for the non‐expert in marine ecology is given in ref. 46, 47. More in depth surveys are presented in ref. 48, 49. Oil destroys the insulating ability of fur‐bearing mammals and the water repellency of a bird's feathers. In cold waters birds and mammals will die from hypothermia. Many birds and animals also ingest oil when they try to clean themselves, which can poison them. Fish and shellfish may not be exposed immediately, but can come into contact with oil if it is mixed into the water column. When exposed to chronic oil concentration, adult fish may experience reduced growth, enlarged livers, changes in heart and respiration rates, fin erosion, and reproduction impairment. Oil also adversely affects eggs and larval survival. Acute toxicity results in death of exposed fishes.

Reports concerning ecological impact from tanker accidents mostly report the damage done to birds and marine mammals. In the Amoco Cadiz accident several plant species, marine mammals and other crustaceans were affected due to the spill and an estimated 300 000 sea birds were killed. The Torrey Canyon disaster caused the death of around 15 000 sea birds, along with huge numbers of marine organisms. Responders after the Exxon Valdez catastrophe found carcasses of more than 35 000 birds and 1 000 sea otters, which was considered to be a fraction of the animal death toll because carcasses typically sink to the seabed. It is estimated that between 350 000 and 600 000 seabirds, 2800 sea otters, 300 harbor seals, 150 bald eagles, up to 22 killer whales died along with billions of salmon and herring eggs. It is estimated that the Braer tanker accident caused death of more than 6500 oiled seabirds.

The ecological impacts of the Deepwater Horizon disaster have been relatively well studied, compared to many other accidents. Acute impacts can be seen more clearly, like oiled birds and marine mammals. The long term chronic consequences are much more difficult to assess, since they require monitoring over a long period of time. A group of 14 research institutions are working in a program called Ecosystem Impacts of Oil and Gas Inputs to the Gulf^50^ to monitor the long‐term effects and mechanisms of ecosystem recovery from the Deepwater Horizon blowout.

The oil spill has caused deleterious effects in the ecosystems. Some have not yet fully recovered. The accident has also reminded the need for more knowledge on baseline chemistry data for deep‐ocean ecosystems. Without such information it is extremely difficult to assess the impacts from oil activities in organisms. In October 2016 a movie called Deepwater Horizon has been released and will probably reignite the attention to the disaster in 2010.^51^ Researchers at Florida International University (FIU) have been uncovering the far‐reaching environmental damage done to the Gulf of Mexico from the oil spill.^52^

### 4.3 Impact on Freshwater Ecology in the Niger Delta

The impact on freshwater resources of oil and oil related activities and chemicals are exemplified by the leakages in the Niger Delta. Oil contaminants have been detected in virtually all water media used by the public for different purposes, from surface water to rainwater and from hand‐dug wells to borehole groundwater sites.^15^, ^23^, ^53^, ^54^, ^55^, ^56^ The social and economic consequences have been devastating for the local population and are further discussed in the next section.

Barium is a heavy metal used by the oil industry in drilling mud, which is then often left in the mud pits around wellheads or dumped offshore. Water quality of groundwater in the Central Niger Delta was studied and high concentrations of Barium, above the WHO permissible limits, were recorded in all the water samples analyzed.^57^, ^58^ They attributed the high barium content in the groundwater aquifers to discharges of drilling waste and the erosion of weathered rock, which leached down from the surface to groundwater systems. Excessive uptake of water‐soluble barium may cause a person to experience vomiting, abdominal cramps, diarrhea, difficulties in breathing, increased or decreased blood pressure, numbness around the face, and muscle weakness.^59^ The big fisted swimming crab, *Callinectes Amnicola*, is one of the commonest edible crabs fished for in Niger Delta wetlands. *C. Amnicola* exposed to drilling mud (EDC‐99‐DW) had incremental tissue accumulation of barium with increasing concentration of the latter, up to 5,727 mg kg^–1^.^60^ Histological consequences of the drilling mud on the crab included irregular tissue shape, macrophages, inflammatory cells and basophilic spots.

Nigeria brands of crude oil are known to contain relatively high concentrations of some heavy metals.^61^ For Nigeria's dominant ‘sweet’ crude oil, Bonny Light, the associated heavy metals occur in the order nickel>vanadium>cadmium>copper>lead.^62^ High concentrations of barium, lead and cadmium, above WHO permissible limits^58^ were recorded in borehole and open hand‐dug well waters in the Western Niger Delta,^63^ which the authors attributed to oil exploration and processing activities. They studied levels of heavy metals–cadmium, chromium, copper, iron, nickel and lead–analyzed in the River Ijana, which receives petroleum effluents from the Warri Refinery, Western Niger Delta and reported concentrations generally above WHO standards recommended for surface waters.

Consequences of oil spills in the Niger Delta are exemplified by the Bodo Creek case, Ogoniland, eastern Niger Delta.^64^ Over 65% of Bodo features a network of brackish water creeks, mangrove swamps and pockets of island forest.^65^ Bodo is a rural coastal town with a population of over 49 000 people. The majority of its inhabitants are subsistence fisherfolks and farmers. Two spills in 2008–2009 from the Trans‐Niger pipeline were leaking out 80 000–95 000 *m* ^3^ in the Bodo Creek area. It has been estimated that 1000 hectares of mangroves were destroyed by the spills and additional 5000 hectares were chronically impacted, the largest loss and damage to mangroves by oil the world has ever seen.^66^, ^67^

Relying on empirical data on the water quality of Bodo Creek before the 2008 spills.^65^, ^68^, ^69^, ^70^, ^71^ impacts of the spills on the creek ecology and biodiversity was estimated.^31^ The locations having been studied before the spills then were resampled after the spills in 2011. A 91% defaunation of the intertidal macrozoobenthos (animals >0.5 mm that live in sediment) was recorded.^31^ In particular, the West African lucinid bivalve, *Keletistes Rhizoecus*, known to be endemic in the Niger Delta had distinctly constituted an average 96.5% of the infauna community abundance pre‐spill at the Sivibilagbara mangrove swamp in Bodo Creek, having monthly density of 333 to 1583 m^–2^ pre‐spill. It was absent post‐spill. Fish and shellfish species such as mullets, snappers, cichlids, tilapia, croakers, grouper, intertidal mudskippers and bonga fish, swimming crab, shrimps, oyster, periwinkle, bloody cockle, whelk and dog whelk that provided an extensive economic, food and cultural resource base for the pre‐spill Bodo community were heavily impacted.^64^, ^72^

Pre‐spill and post‐spill metal concentrations in surface water and sediments in Bodo Creek were compared.^73^ They reported significant increase in metal (zinc, lead and copper) loads post‐spill. A follow‐up study^74^ on metal concentrations in water and sediment in Bodo Creek also reported concentrations of magnesium, iron, copper, zinc, chromium, lead and cobolt as being higher than the World Health Organization^58^ recommended permissible levels for surface and drinking water.

## 5 Economic and Social Consequences

Comparing the two major oil spill locations, the Mexican Gulf and the Niger Delta, there are certainly many similarities in the ecological consequences. However, the economic and social aftermaths depend significantly on the country and on the media attention of the tragedies.

### 5.1 Mexican Gulf

The social and economic consequences of the Deepwater Horizon accident were huge, affecting the fishing industry, local fishermen and business activities along the Gulf coastline, lost property values and many other after‐effects. The effects on human health were documented in a National Academies Press workshop in 2010.^75^ In January 2011 the White House oil spill commission released its final report on the accident in 2010.^76^ BP, Halliburton, and Transocean were all, in different ways, blamed for making a series of cost‐cutting decisions and the lack of a system to ensure well safety. The report also concluded that “the root causes are systemic and, absent significant reform in both industry practices and Government policies, might well recur”. As of February 2013, criminal and civil settlements and payments to a trust fund had cost BP US$42.2 billion, where it is expected that people affected and property damaged by the accident would be appropriately reimbursed.

### 5.2 Niger Delta

Oil has generated an estimated US$600 billion to the Nigerian government since the 1960s.^77^ Despite this, the majority of the Niger Delta's population lives in poverty with substandard social infrastructure and increasing levels of oil contamination of their natural resource and livelihoods dependent ecosystems.^23^ The poor oil spill management regime is clearly demonstrated by the oil spills in the Bodo Creek area. A fault in the 24'' (600 mm) diameter pipeline on August 28, 2008 resulted in a significant oil spill into the Bodo Creek. The oil company claims that they were informed of the leak not until early October 2008. Even then it took the oil company over a month to repair the weld defect in the pipeline. A second major spill occurred in the same creek on December 7, 2008 and was also the result of equipment failure. It was not capped until February 19, 2009 during which time even greater damage was inflicted upon the Bodo Creek as crude oil pumped into the creeks and mangrove swamps over a period of two months. Since the oil spills the over 13 000 fisher folks from the Bodo community have been unable to continue fishing in the Bodo Creek.^25^, ^64^

The impact of the Bodo oil spills on the environment, water quality, local income generation, employment, livelihood structures and community development with particular focus on fishing and ancillary industries of the user population is documented in detail.^64^ Examinations were made on the effects of the Bodo oil spills on indigenous practices and cultural human rights of the user population, including impacts on community recreation, traditional experience of childhood, beliefs and rituals, impact on the elderly, fishing practices, cooperative weeding, migration, etc.^78^

The life expectancy in the rural communities of the Niger Delta, half of which have no access to clean water, has fallen to little more than 40 years over the past two generations. Locals blame the oil that pollutes their land and can scarcely believe the contrast with the steps taken by BP and the U.S. Government to try to stop the Mexican Gulf oil leak and to protect the Louisiana shoreline from pollution. Ben Ikari, a member of the Ogoni people said that “if the (Mexican) Gulf accident had happened in Nigeria neither the Government nor the Company would have paid much attention. This kind of spill happens all the time in the Delta.”

Following the spills around Bodo the price of fish, a local staple food, rose as much as tenfold.^79^ Oil spills in the Ogoniland region have also contaminated local drinking water sources, seeping into groundwater, according to United Nations Environment Programme.^23^ Toxins found by UNEP in the wider Ogoniland area's drinking water include benzene, which is thought to cause cancer.

In January 2015 Shell agreed to pay the Bodo community £55 million for the oil spills in 2008. The agreement was the end of a four‐year legal battle. Shell also agreed to a long‐overdue cleanup, but a UN report has said it could take 30 years to properly restore the ruined land, creeks and mangrove swamps in Ogoniland. Moreover, cleanup, remediation and restoration of the heavily impacted Bodo Creek according to international best practice will cost a fortune.

The oil leakages in Nigeria can be put in some perspective by comparison with smaller leakages that have got more media coverage. An oil pipeline leak occurred in 2013 in North Dakota, U.S.^80^ The 20 year old Tesoro pipeline, located under a wheat field, broke and caused about 3000 m^3^ (840 000 gallons) of oil to spill, covering some 7 acres (≈30 000 m^2^) and within the top 3 m of clay soil. The cleanup effort has taken years and has been estimated to cost around US$4 million. A spokesman for the Stakeholder Democracy Network in Lagos, Nigeria, which works to empower communities affected by the oil companies' activities, said: “The response to the spill in the U.S. should serve as a stiff reminder as to how far spill management in Nigeria has drifted from standards across the world.”

## 6 Mitigating Leakage Risks

Efforts are made on a continuous basis to mitigate risks for oil accidents, spills and leakages. Better tanker design, enhanced equipment maintenance as well as improved methods for safer drilling are examples of such efforts. Here we highlight one important line of development: automatic leakage detection. Measurements in pipelines are essential tools to make the transportation of oil safer.

Comparing the cost for the damages, the cost for not detecting a leakage problem, is usually countless times higher than the cost for monitoring and automatic detection. When a leak occurs in a pipeline, an alarm should be automatically initiated and should communicate not only the leak detection but also the location of the leak. The accuracy of the leak location estimation depends among other things on the size of the leakage flow. Also imperfections in a pipe can be identified by inspection tools and corrected before they progress to a leak.^81^

A leak detection system will provide an alarm and display other related data to the pipeline controllers in order to aid in decision‐making. Pipeline leak detection systems are also beneficial because they can enhance productivity and system reliability thanks to reduced downtime and less inspection time. In internally based leak detection systems there are field sensors used for primarily flow rate, pressure or fluid temperature. The sensors are used to monitor internal pipeline parameters. During steady‐state conditions, the flow rate, pressure and temperature in the pipeline are (more or less) constant over time. A leak changes the hydraulics of the pipeline, and therefore changes the pressure or flow readings after some time. Local monitoring of pressure or flow at only one point can therefore provide simple leak detection.

During transient conditions, the hydraulic variables may change rapidly. The changes propagate like waves through the pipeline with the speed of sound of the fluid. Transient conditions occur in a pipeline for example at start‐up, if the pressure at inlet or outlet changes (even if the change is small), or when multiple products are in the pipeline. Gas pipelines are almost always in transient conditions, because gases are compressible. Even in liquid pipelines, transient effects cannot be disregarded most of the time.

The acoustic pressure wave method analyses the pressure waves produced when a leak occurs. When a pipeline wall breakdown takes place, fluid or gas escapes in the form of a high velocity jet. This produces negative pressure waves which propagate in both directions within the pipeline and can be detected and analyzed. The operating principles of the method are based on the significant characteristic of pressure waves to travel over long distances at the speed of sound guided by the pipeline walls. The amplitude of a pressure wave increases with the leak size. Acoustic systems can also be applied to a wide range of fluids and scenarios–above‐ground, buried, subsea, liquids, gas, and also some multiphase fluids. Multiple monitored sections can be defined and integrated according to each application. In some cases the sensors (monitoring points) can be spaced up to 30 or 40 km still keeping good sensitivity.

Another type of leak detection system is based on detection by physical contact (absorption) between the liquid oil and a sense cable located along the pipeline. The cable consists of a braid of semi‐permeable internal conductors protected by a permeable insulating moulded braid. Escaping fluids pass through the external permeable braid and make contact with the internal semi‐permeable conductors. An electrical signal is monitored as a result of the leak.^82^

Externally based leak detection systems also utilize field instrumentation ‐for example infrared radiometers, thermal cameras, vapor sensors, acoustic microphones or fiber‐optic cables to monitor external pipeline parameters. Such systems are highly sensitive and accurate, but system cost and complexity of installation are usually high. Applications are therefore limited to special high‐risk areas, e.g. near rivers or nature‐protection areas.

Several leak detection methods, their advantages and disadvantages have been reviewed^83^, ^84^ and appropriate solutions for different pipeline systems are suggested.

## 7 Conclusion

Oil exploration depends on the availability of water. Crude oil pumping, hydraulic fracturing, as well as oil sand exploration and refining use significant volumes of water. In water scarce areas the availability of water is a particularly sensitive issue. Produced water from oil operations present significant challenges to treat and recover to acceptable quality.

Oil operations are exposed to high risks with large economic and environmental consequences when an accident or a leakage occurs. With an increasing demand for oil and gas the explorations have to rely on increasingly advanced technology to find oil that is less readily reachable. Accidents and spills caused by oil exploration in different parts of the world have been illustrated. In most cases the consequences of spills are directly observed in the water resources. Here we have described some specific episodes of oil accidents and spills in order to illustrate the impact on water quality and ecology. The economic and social aftermaths are often substantial. Transportation of oil via tankers or pipelines presents still another challenge.

Leakages in pipelines and tanker accidents are real threats that can never be eliminated. However, they can be minimized by adequate maintenance practices, early warning leakage sensor systems, careful design of tankers or pipelines and ever improving operating procedures.

The world depends on oil resources for a multitude of uses. Even as alternative energy sources are being developed the reliance on oil will remain very high for many decades. Therefore the environmental consequences of oil exploration, transportation and use have to be carefully scrutinized, in particular with respect to water.

## Conflict of Interest

The authors declare no conflict of interest.
